# Cerebellar morphometric and spectroscopic biomarkers for Machado-Joseph Disease

**DOI:** 10.1186/s40478-022-01329-4

**Published:** 2022-03-19

**Authors:** Catarina Oliveira Miranda, Rui Jorge Nobre, Vitor Hugo Paiva, João Valente Duarte, João Castelhano, Lorena Itatí Petrella, José Sereno, Magda Santana, Sónia Afonso, Cristina Januário, Miguel Castelo-Branco, Luís Pereira de Almeida

**Affiliations:** 1 Center for Neuroscience and Cell Biology (CNC), University of Coimbra (UC), Rua Larga, Pólo I, 1º andar, 3004-504 Coimbra, Portugal; 2 Center for Innovative Biomedicine and Biotechnology (CIBB), Faculdade de Medicina, UC, Rua Larga, Pólo I, 1º andar, 3004-504 Coimbra, Portugal; 3 Institute for Interdisciplinary Research (III), UC, Casa Costa Alemão - Pólo II, Rua Dom Francisco de Lemos, 3030-789 Coimbra, Portugal; 4 ViraVector, Viral Vector for Gene Transfer Core Facility, UC, Coimbra, Portugal; 5 Marine and Environmental Sciences Centre, Department of Life Sciences, UC, 3004-517 Coimbra, Portugal; 6 Coimbra Institute for Biomedical Imaging and Translational Research (CIBIT), UC, Edifício Do ICNAS, Polo 3, Azinhaga de Santa Comba, 3000-548 Coimbra, Portugal; 7 Institute of Nuclear Science Applied To Health (ICNAS), UC, Polo 3, Azinhaga de Santa Comba, 3000-548 Coimbra, Portugal; 8 Institute for Biomedical Imaging and Life Sciences (IBILI), UC, Polo 3, Azinhaga de Santa Comba, 3000-548 Coimbra, Portugal; 9 Department of Electrical and Computer Engineering, Faculty of Science and Technology, UC, Polo 2, 3030-290 Coimbra, Portugal; 10 Neurology Department, Coimbra University Hospital Center, Praceta Prof. Mota Pinto, 3000-075 Coimbra, Portugal; 11 Faculty of Medicine, UC, Rua Larga 2, 3000-370 Coimbra, Portugal; 12 Faculty of Pharmacy, UC, Polo 3, Azinhaga de Santa Comba, 3000-548 Coimbra, Portugal

**Keywords:** Machado-Joseph disease (MJD), Spinocerebellar ataxia type 3 (SCA3), Magnetic resonance imaging (MRI), Proton magnetic resonance spectroscopy (^1^H-MRS) biomarkers, Motor performance

## Abstract

Machado-Joseph disease (MJD) or Spinocerebellar ataxia type 3 (SCA3) is the most common form of dominant SCA worldwide. Magnetic Resonance Imaging (MRI) and Proton Magnetic Resonance Spectroscopy (^1^H-MRS) provide promising non-invasive diagnostic and follow-up tools, also serving to evaluate therapies efficacy. However, pre-clinical studies showing relationship between MRI-MRS based biomarkers and functional performance are missing, which hampers an efficient clinical translation of therapeutics. This study assessed motor behaviour, neurochemical profiles, and morphometry of the cerebellum of MJD transgenic mice and patients aiming at establishing magnetic-resonance-based biomarkers. ^1^H-MRS and structural MRI measurements of MJD transgenic mice were performed with a 9.4 Tesla scanner, correlated with motor performance on rotarod and compared with data collected from human patients. We found decreased cerebellar white and grey matter and enlargement of the fourth ventricle in both MJD mice and human patients as compared to controls. *N*-acetylaspartate (NAA), NAA + *N*-acetylaspartylglutamate (NAA + NAAG), Glutamate, and Taurine, were significantly decreased in MJD mouse cerebellum regardless of age, whereas myo-Inositol (Ins) was increased at early time-points. Lower neurochemical ratios levels (NAA/Ins and NAA/total Choline), previously correlated with worse clinical status in SCAs, were also observed in MJD mice cerebella. NAA, NAA + NAAG, Glutamate, and Taurine were also positively correlated with MJD mice motor performance. Importantly, these ^1^H-MRS results were largely analogous to those found for MJD in human studies and in our pilot data in human patients. We have established a magnetic resonance-based biomarker approach to monitor novel therapies in preclinical studies and human clinical trials.

**Supplementary Information:**

The online version contains supplementary material available at 10.1186/s40478-022-01329-4.

## Introduction

Spinocerebellar ataxias are a group of autosomal dominant, genetically inherited neurodegenerative disorders characterized by progressive damage of the cerebellum and cerebellar interconnections. Among these, spinocerebellar ataxia type-3 (SCA3) or Machado-Joseph Disease (MJD) is the most common worldwide. MJD is caused by over-repetition of the triplet CAG in *ATXN3/MJD1* gene, which translates into an expanded polyglutamine (polyQ) tract within the mutated protein ataxin-3 [1]. Mutant ataxin-3 accumulates in the form of neuronal intranuclear inclusions and simultaneously impairs several cellular pathways, culminating in neurodegeneration [2]. Multiple brain regions can be affected, namely the cerebellum, the basal ganglia, the brainstem, and the upper segment of the spinal cord [3–7]. Clinically, patients can present severe ataxia, dysarthria, dysphagia, lack of eye movement control, bulging eyes, diplopia, dystonia, spasticity and Parkinsonism, ending up in premature death [6, 8–10]. There is no therapy that can stop disease progression. However, promising therapeutic approaches are under development [11–18] for whose validation there is an urgent need of biomarkers.

Magnetic Resonance Imaging (MRI) and Proton Magnetic Resonance Spectroscopy (^1^H-MRS) are elegant non-invasive tools performed in the same scanning equipment that allow assessment of structural and neurochemical alterations throughout time and can serve to validate therapies under evaluation. MRI/^1^H-MRS techniques have been previously used in MJD patients. Besides volumetric assessments, specific metabolites (or ratios) such as *N*-acetylaspartate have been established to directly vary with duration of disease, age of onset, number of CAG repeats or SARA score, providing a promising tool in determining disease severity [19–22]. However, studies in rodent models of MJD have been very rare [23, 24]. A single study was performed so far in SCA3/MJD models, where the authors correlated the neurochemical biomarkers in homozygous YACMJD84.2 (Q84/Q84) and hemizygous CMVMJD135 (Q135) mice with the levels of Neurofilaments (NFL) and MBP measured in cerebellar lysates from a subset of mice and patients with SCA3 [24]. Here, we correlate for the first time in transgenic mice and MJD patients, MRI/MRS data analysis with motor impairments, known to be a crucial hallmark of this disease.

Therefore, the aim of the present study was to identify cerebellar morphometric and spectroscopic biomarkers picturing the phenotypic status of another MJD transgenic mouse model through in vivo MRI/^1^H-MRS analysis and further evaluate the translational potential of these biomarkers by assessing their values in human MJD patients. Moreover, it also addresses structural MRI findings (both in mice and MJD patients) further establishing a clear correspondence between cerebellar volumetric parameters in this transgenic animal model and patients, which enhances the clinical relevance of this SCA3/MJD model. We propose that such MRI/^1^H-MRS parameters could be used in these and other MJD animal models, as well as in human patients, to evaluate the efficacy of therapies.

## Methods

### Animals

A total of 57 animals of 2, 4 or 16 months old were used in this study: twenty-nine MJD transgenic mice (C57BL/6 background) expressing the N-terminal truncated human ataxin-3 with 69 glutamine repeats (Tg-ATXN3-69Q) [25] and twenty-eight wild type (WT) controls. Animals randomly obtained from our colony were distributed in the following groups: control, 2 months-old (n = 14, 6 male + 8 female); MJD Tg, 2 months-old (n = 14, 4 male + 10 female); control, 4 months-old (n = 7 female); MJD Tg, 4 months-old (n = 9 female); control, 16 months-old (n = 7, 4 male + 3 female); MJD Tg, 16 months-old (n = 6, 5 female + 1 male). Of note, to reduce the total number of animals and fulfill the reduction objective of the "3Rs principle", the animals used in the present study were used in other studies for different purposes.

Animals were housed in groups (2–5 per cage, depending on cohort study) in plastic cages (365 × 207 × 140 mm) with food and water ad libitum, and maintained on a 12-h light/dark cycle at a room with constant temperature (22 ± 2 °C) and humidity (55 ± 15%). The animals were allowed 1 week of acclimation to the surroundings before the beginning of the behavioural tests or MRI/^1^H-MRS acquisitions. Physical state of animals was evaluated daily, and weight measured every week.

All animal experiments were carried out in accordance with the European Community Council Directive (86/609/EEC) for the care and use of laboratory animals and were previously approved by the Responsible Organization for the Animals Welfare of the Faculty of Medicine and Center for Neuroscience and Cell Biology of the University of Coimbra (ORBEA and FMUC/CNC, Coimbra, Portugal) (ORBEA_66_2015/22062015). All researchers were certified to perform the experiments with animals (Felasa-certified course and Direcção Geral de Veterinária, Lisbon, Portugal) (DGAV: 0421/000/000/2015).

### Subjects

Patients enrolled in this study were followed in the Neurology Department of *Centro Hospitalar e Universitário de Coimbra—CHUC* and had a confirmed molecular diagnosis of MJD (carriers of a CAG repeat expansion in *ATXN3* gene). Controls were recruited from the community. Control participants had no personal or family history of neurological or psychiatric diseases. Neurological examination was performed by a trained neurologist. Oculomotor abnormalities, pyramidal, extrapyramidal, and peripheral nerve involvement were evaluated, and the severity of ataxia was determined by applying the Scale for the Assessment and Rating of Ataxia (SARA) [26]. On subjects belonging to families that were previously followed in our department, age of onset was considered when patients became symptomatic (SARA score superior to 4); non-ataxic symptoms were questioned and evaluated particularly considering oculomotor abnormalities and peripheral nerve involvement. Patient 16 (Additional file 10: Supplemental table I) had diplopia first noticed when reading, and on examination he had a gaze-evoked niystagmus, abnormal saccades, impaired vestibulo-ocular reflex. These symptoms clearly mark the onset of disease (the conversion), despite having a small ataxic score. Considering patient 12 (Additional file 10: Supplemental table I), she had a confirmed peripheral neuropathy, diagnosed when she started complaint of lower limbs pain about 2 years before gait ataxia. The same was observed for patient 2 (Additional file 10: Supplemental table I), a female with oculomotor abnormalities starting 3 years before the first symptoms of ataxia. If patients were not previously followed in our department, age of onset was determined based on patients’ motor and ataxic reports. Included patients were assessed using a standardized protocol, including demographic, clinical and a cognitive evaluation. We used The Montreal Cognitive Assessment (MoCA) (Portuguese version) as the screening test for dementia. Patients with severe hypertension, diabetes or mild cognitive impairment were excluded from this study.

In the cross-sectional morphometry study were included 16 (9 male/7 female) MJD (average of age: MEAN ± SEM = 41.15 ± 3.04) and 18 (10 male/8 female) healthy controls (CNT, average of age: MEAN ± SEM = 33.19 ± 2.35). Exploratory MRS acquisitions were performed in 2 MJD patients (2 females of 32 and 56 years) and 2 controls (2 females of 33 and 57 years). Patients with availability and support to realize this exam were invited to participate. This study was approved by the Ethics Committee of the Faculty of Medicine of University of Coimbra—FMUC, and all participants signed an informed consent before data collection.

### MRI/MRS acquisitions in animals

In vivo image acquisitions were conducted with a 9.4 T magnetic resonance small animal scanner (BioSpec 94/20, Bruker Corporation, Ettlingen, Germany) with a standard cross coil setup using a volume coil for excitation (with 86/112 mm of inner/outer diameter, respectively) and quadrature mouse surface coil for signal detection, at the Institute for Nuclear Sciences Applied to Health (ICNAS), University of Coimbra.

Blind assessments were carried out throughout the study. For volumetric analyses, T2-weighted images were acquired in coronal planes using a RARE sequence: TR = 2500 ms; TE = 33 ms; 6 averages; pixel size of 0.0781 mm × 0.0781 mm and slice thickness of 0.5 mm without spacing between slices (total head volume 256 pixels × 256 pixels × 34 slices), and a total scan time of 20 min. The cerebellum segmentation was carried out with custom made software developed in MATLAB programming language. The first step contemplates the correction of magnetic field inhomogeneities generated from the surface coil; the correction was implemented using intensity curves obtained from a homogeneous phantom and acquired with the same coil and system configuration. Then, the intensities of the images were normalized between 0–10,000 Gy levels, after outliers’ exclusion (1%). The segmentation itself consisted of four steps: (1) the manual segmentation of the whole cerebellums; (2) the automatic segmentation of cerebellar white matter (WM) and gray matter (GM) based on contours detection using a Laplacian of Gaussian filter (Additional file 1: Supp. Figure 1); (3) the regions corresponding to WM or GM were manually selected; (4) the corresponding volumes were obtained by multiplying the number of voxels belonging to each region by the voxel size.

^1^H-MRS data were collected on a volume of interest placed on the cerebellum (VOI: 4.0 mm × 1.1 mm × 1.0 mm; V_t_ = 4,40 mm^3^), using multi-slice rapid acquisition and relaxation enhancement (RARE) images, in three orthogonal directions (axial, coronal and sagittal) with the following parameters: TR = 2500 ms, TE = 33 ms, matrix size (256 × 256), field of view = (20 × 20) mm^2^, 22 slices, slice thickness = 0.5 mm, 1 average and scanning time = 1 min and 20 s. 

### MRI/MRS acquisition—Humans

Data were collected with a Siemens Magnetom TIM Trio 3 Tesla scanner (Siemens, Munich, Germany) with a phased array 12-channel birdcage head coil. We acquired a 3D anatomical T1-weighted MPRAGE (magnetization-prepared rapid gradient echo) magnetic resonance imaging pulse sequence (TR 2530 ms; TE 3.42 ms; TI = 1100 ms; flip angle 7°; 160 single-shot interleaved slices with no gap with isotropic voxel size 1 × 1 × 1 mm; FOV 256 mm) of all participants. ^1^H-MRS data were collected on a volume of interest placed on the cerebellum using a PRESS sequence with the following parameters (both for unsuppressed or suppressed water signal): TR = 2000 ms, TE = 35 ms, number of averages = 160 (16 for NWS), flip angle = 90º, Bandwidth = 1200 Hz. After data processing, peaks were quantified using LCModel software package (Stephen Provencher Inc., Oakville, Canada; Provencher 1993), adapted.

### Spectroscopy analysis in animals

Data were saved as free induction decay (FIDs), corrected for the frequency drift and corrected for residual eddy current effects using the reference water signal. Then ^1^H NMR peak concentrations for major metabolites (e.g., NAA, GABA, Tau and Glx) were analyzed using LCModel software package (Stephen Provencher Inc., Oakville, Canada; Provencher 1993) and results are given relative to water content in tissue, as described in previous studies from our team [27, 28]. Briefly, the LCModel analysis calculates the best fit to the acquired spectrum as a linear combination of model, basis set of brain metabolites. The Cramer-Rao lower bound (CRLB) provided by LCModel was used as a measure of the reliability and metabolite concentrations with Cramer-Rao lower bound higher than 24% were not included in the analysis. Spectral quality was evaluated by visual inspection of the signal to noise ratio that were provided by LCModel.

### Image processing and estimation of volumetric measures—Humans

All images were processed and analyzed using the CAT12 toolbox (C. Gaser, Structural Brain Mapping Group, Jena University Hospital, Jena, Germany; http://dbm.neuro.uni-jena.de/cat/) implemented in SPM12 (Wellcome Trust Centre for Neuroimaging; http://www.fil.ion.ucl.ac. uk/spm/software/spm12/). CAT12 served as the platform for all the analyses, as it offers processing pipelines for volumetric global and local measures (using atlas-defined ROIs). For processing- and analysis-steps, pre-set parameters in accordance with standard protocols (http://www.neuro.uni-jena.de/cat12/CAT12-Manual.pdf) were used. Processing also included a two-step quality assurance: first, all images were visually inspected for artefacts (prior to pre-processing); secondly, all underwent a statistical quality control for inter-subject homogeneity and overall image quality as included in the CAT12 toolbox (“check homogeneity” function) after segmentation. This second step again included a visual inspection procedure for potential newly introduced artefacts.

For tissue segmentation, briefly, all 3D T1-weighted MRI scans are normalized using a affine followed by non-linear registration, corrected for bias field in homogeneities, and then segmented into grey matter (GM), white matter (WM) and cerebrospinal fluid (CSF) using the Diffeomorphic Anatomic Registration Through Exponentiated Lie algebra algorithm (DARTEL) to normalize the segmented scans into a standard MNI space. During this procedure the brain is also automatically parcellated into the left and right hemisphere, subcortical areas, and the cerebellum. The total intracranial volume (TIV, here referred in the total volume of the cerebellum) was calculated as the sum of the GM, WM, and CSF volumes.

### Spectroscopy analysis in Humans

The voxel was placed to cover the cerebellum as shown in the Fig. 2C and the voxel dimension was 30 × 30 × 30 mm^3^. Data were analyzed using LCModel software package. The LCModel analysis calculates the best fit for the acquired spectrum as a linear combination of model, basis set of brain metabolites. The Cramer-Rao lower bound (CRLB) criterion was used as a measure of the reliability of the apparent quantification of the metabolite concentrations. We followed a protocol we reported previously [27, 28], and metabolite concentrations with CRLB higher than 24% were not included in the analysis. Results were given relative to water content in tissue. Spectral quality was evaluated by visual inspection.

### Behavioural assessment

Fore and hind limb motor coordination as well as balance, were evaluated in two and 4-month-old Tg-ATXN3-69Q MJD mice by rotarod (Letica Scientific Instruments). For rotarod analysis, animals were placed on the rotarod at a constant (5 rpm for a maximum of 5 min, stationary rotarod) or an accelerated speed (from 4 to 40 rpm in 5 min, accelerated rotarod), and the latency to fall (the amount of time they could stand in the rotated wheel) was recorded. Mice were subjected to 4 trials for each test at each time point, with a minimum of 15 min’ rest between trials. Older mice (16-month-old) were not included as MJD mice already presented some loss of strength in their limbs.

### Statistics

In the behavioural tests, the effect of group (e.g., WT vs. MJD mice) on behavioural response variables (latency to fall from a rotarod) was assessed through Unpaired t test with Welch’s correction, to identify significant differences among groups. A Principal Component Analysis was run to reduce the variability of the metabolites’ values of WT and MJD mice into two main axis of variation (PC1 and PC2) and to identify the changes in concentration of metabolites more associated with each study group. PCA was run using the function *prcomp* from the R package *stats* (Team., 2018). A Permutational Analysis of Variance (PERMANOVA) was used to compare means of the concentration of metabolites (e.g. GABA) between WT and MJD mice, using the function *adonis* from the *Vegan* R package (Oksanen, 2019). These statistical analyses were carried out in R statistical software (Version 4.00). Variables were tested for normality (Q-Q plots) and homogeneity (Cleveland dotplots) before each statistical test and transformed when needed.

Linear regression tests were used to study the relationship between behavioural tests results (e.g., latency to fall from a rotarod) with diverse metabolic compounds measured by ^1^H-MRS, (Graph Prism, version 8.1.2).

For human data, multiple regression models were used to test the effect of group, age, gender, and their interaction on grey matter (GM), white matter (WM), cerebrospinal fluid (CSF) and total cerebellar volumes. The covariates age and gender were only kept in the models when they had a significant effect in explaining data variability. On MJD patients, multiple regression models were also used to test the effects of age of onset, disease duration, number of CAG repeats in disease allele, and SARA score on GM, WM, CSF, and total cerebellar volumes. Prior to running multiple regression models, correlations between independent variables were tested. When two variables were highly correlated (r > 0.7), only the variable with the lowest Akaike Information Criteria (AIC) and *p* value in a univariate model was selected, to avoid high collinearity (see Additional file 2: Supp. Figure 2). According to these criteria, the effects of disease onset and SARA score were tested in our variables of interest. Bonferroni adjustments were applied for p-values of terms within multiple regression models. Statistical analyses were carried out in R statistical software (Version 4.00).

All analyses were performed assuming a significance level of P ≤ 0.05.

## Results

### I—MJD transgenic mice (Tg-ATXN-3-69Q) and MJD patients show similar structural/anatomical cerebellar alterations

In the present study, a total of 28 WT and 29 MJD mice were analysed, including 2, 4, and 16-month-old mice, as detailed (Additional file 3: Supp. Figure 3). The cerebellum was the selected brain region for analysis, since it is one of the most affected brain regions in MJD patients, and the most affected in this mouse model [25].

**Fig. 1:**
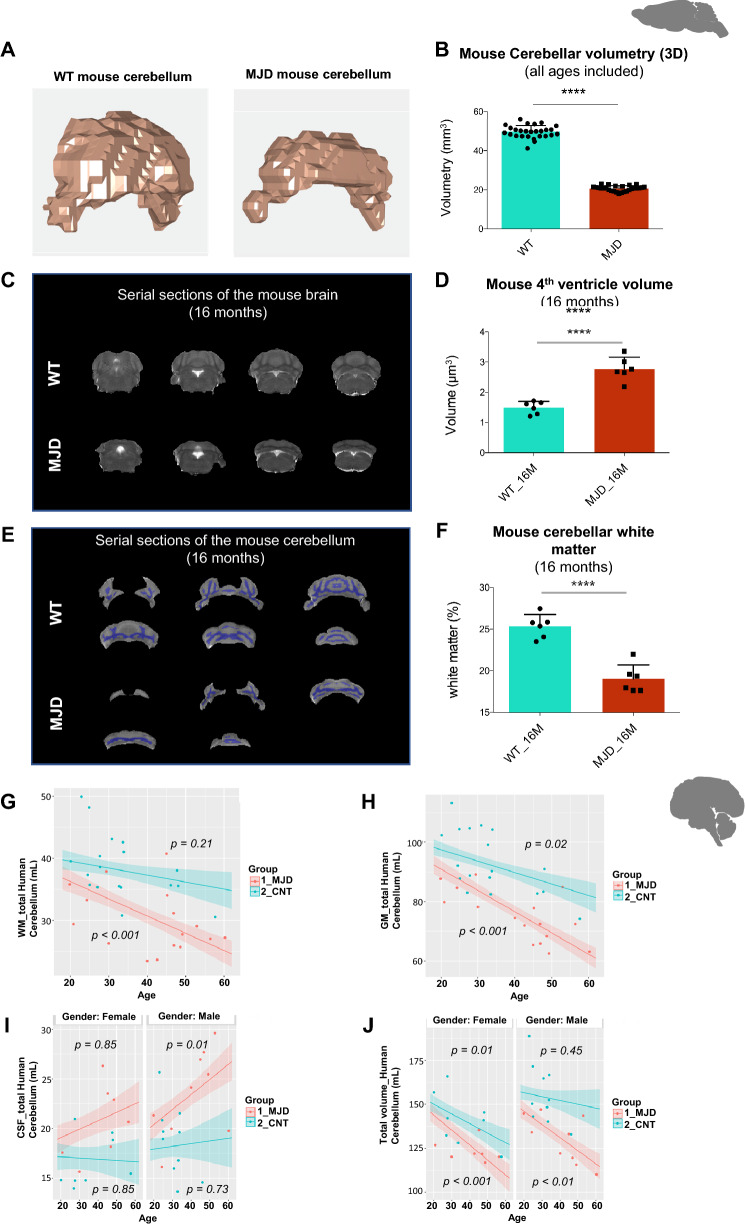
Cerebellar structural/anatomical alterations in MJD mice and patients. **a** Representative 3D images of the cerebellum of wild type (WT) and Tg-ATXN3-69Q (MJD) mice. **b** Cerebellar volumetry of WT (n = 27) and MJD (n = 27) mice (all ages included), obtained through the analyses of cerebellar images pre-processed and segmented in Matlab R2012b. **c** Illustrative serial sections of the brain of a WT (up) and an MJD (bottom) mouse with 16 months, showing the space occupied by the 4th ventricle (white structure). **d** Fourth ventricle volume of WT (n = 6) and MJD mice (n = 6) at 16 months obtained through the analyses of cerebellar images pre-processed and segmented in Matlab R2012b. **e** Illustrative serial images of the cerebellum of a WT (up) and an MJD (bottom) mouse at 16 months, showing cerebellar white matter (purple structure). **f** Cerebellar white matter in WT (n = 6) and MJD mice (n = 6) was obtained through the analysis of cerebellar images pre-processed and segmented in Matlab R2012b. Multiple regressions to test the effect of group (MJD patients, n = 16 *vs*. CNT (healthy individuals), n = 18) in WM (**g**), GM (**h**), CSF (**i**) and the sum of those volumes (mL) (**j**) in the whole cerebellum. The covariates age and gender were only kept in the models when they had a significant effect in explaining data variability. From a–f: unpaired t test with Welch’s correction was used to test for differences among groups. Multiple regressions were performed from g–j. ** p < 0.01; ****p < *0.0001. Abbreviations: WM—white matter; GM—grey matter; CSF—cerebrospinal fluid; CNT—controls; MJD—Machado-Joseph disease patients. Image created by me or a co-author*

We started this work by determining cerebellar 3D volumetry of MJD mice when compared to controls by MRI (Fig. 1A). This assessment is more accurate than other indirect techniques (e.g., immunohistochemistry), as it allows in vivo determination, keeping all the anatomical structures intact and avoiding artifacts associated with fixation, cryopreservation, sectioning, and histochemical processing. As expected, a robust decrease (2.39-fold) of the cerebellar volume was found in MJD mice when compared to WT animals, regardless of age (WT: 49.62 ± 0.6140, n = 27 vs MJD: 20.73 ± 0.2684, n = 27; p < 0.0001, *unpaired t test with Welch´s correction*; Fig. 1B). A cross-sectional analysis also showed that this transgenic mouse model has deep loss of the cerebellar volume at an early age with no significant changes over time. (Additional file 4: Supp. Figure 4).

Other morphometric alterations such as the fourth ventricle, the volume corresponding to the pericerebellar CSF spaces, and grey and white matter content in the cerebellum were evaluated by MRI. Similarly to what has been reported in humans MJD patients [29, 30], the volume of the fourth ventricle was highly enlarged in 16-month-old MJD mice when compared to age-matched WT mice (1.85-fold increase; WT: 1.491 ± 0.08530, n = 6 vs MJD: 2.772 ± 0.1603, n = 6; p < 0.0001, Fig. 1C and D) and the white matter content in the cerebellum was substantially decreased (1.33-fold decrease; WT: 25.35 ± 0.5745, n = 6 vs MJD: 19.03 ± 0.6892, n = 6; p < 0.0001, Fig. 1E and F).

To validate the use of these anatomical biomarkers in future clinical studies, results were complemented with data collected from a cohort of sixteen MJD patients and eighteen controls (healthy individuals), whose clinical and etiological information is summarized in (Additional file 10: Supp. Table 1).

Volumetric measurements of WM, GM and cerebellar CSF (volume corresponding to the CSF inside the ROI, i.e., the pericerebellar CSF spaces) were evaluated and compared among groups, where age and gender were used as covariates (Fig. 1G–J).

The volume of WM_totalCbe decreased with age in MJD patients (*β* ± SE: – 0.28 ± 0.07, t = 3.82, p < 0.001), but not in controls (*β* ± SE: − 0.11 ± 0.09, t = 1.27, p = 0.21) (Fig. 1G). The decrease in GM_totalCbe volumetry with age was steepest in MJD patients (*β* ± SE: − 0.72 ± 0.12, t = 5.99, p < 0.001) when compared to controls (− 0.39 ± 0.15, t = 2.64, p = 0.02) (Fig. 1H), denoting that the disease has a strong impact in cerebellar GM and WM volume.

CSF_totalCbe volume increased significantly with age in male MJD patients (*β* ± SE: 0.15 ± 0.06, t = 2.69, p < 0.05), but not in females (*β* ± SE: 0.08 ± 0.06, t = 1.45, p = 0.85) or controls (Males, *β* ± SE: 0.03 ± 0.08, t = 0.35, p = 0.73; Females, *β* ± SE: − 0.01 ± 0.07, t = 0.19, p = 0.85) (Fig. 1I). These results were in accordance with the volumetric measurements done in Tg-ATXN-3-69Q mice and with previous reports in human patients [29, 30]. Finally, the total volume of the cerebellum (GM + WM + CSF, TotalCbeVolume) decreased significantly in female MJD patients (*β* ± SE: − 0.85 ± 0.22, t = 3.89, p < 0.001) and male (*β* ± SE: − 0.74 ± 0.21, t = 3.54, p < 0.01), but also in female controls (*β* ± SE: − 0.55 ± 0.24, t = 3.54, p < 0.05), while in control males it did not decrease significantly (*β* ± SE: − 0.22 ± 0.29, t = 0.76, p = 0.45). As shown by the estimate values, the decrease was steepest in MJD patients when compared to controls (both genders) and within MJD group, in females as compared to males, indicating that this parameter is highly influenced by the disease with age, but also by the gender (Fig. 1J).

**Fig. 2:**
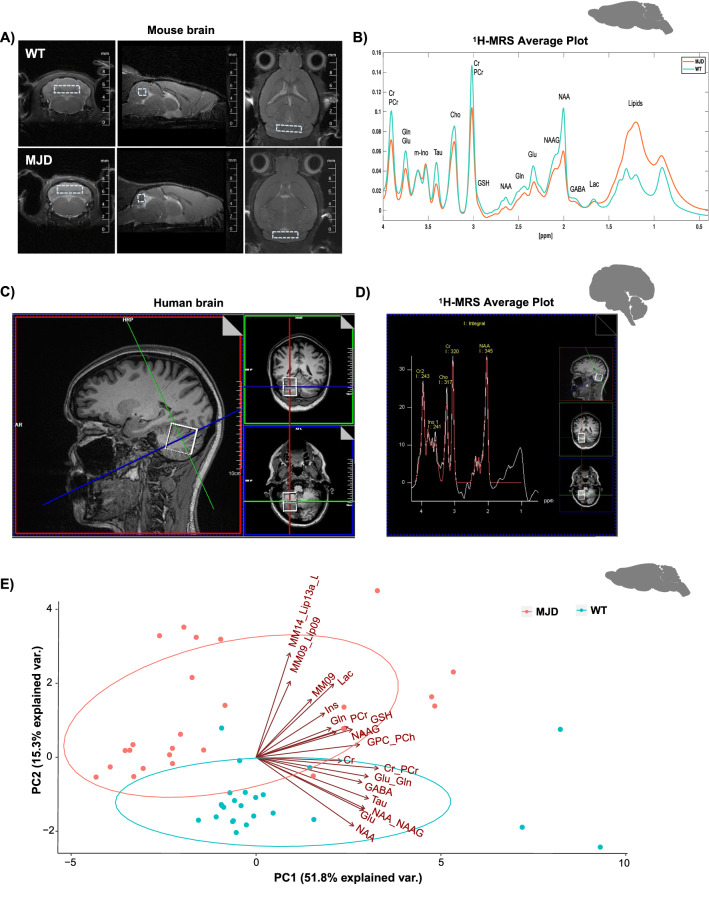
Cerebellar Proton Magnetic Resonance Spectroscopy assessed in MJD mice and patients. **a** Representative images of the cerebellum of wild type (WT) and Tg-ATXN3-69Q (MJD) mice showing voxel localization. **b** Proton magnetic resonance spectroscopy (^1^H-MRS) average plots of WT and MJD mice (all ages included). **c** Representative images of the human brain showing voxel localization. **d** Proton magnetic resonance spectroscopy (^1^H-MRS) average plots of MJD patients and controls. **e** Principal Component Analysis (PCA) of the neurometabolites analysed by ^1^H-MRS in WT and MJD mice. PC1 and PC2 explained 51.8 and 15.3% of the variance in the data, respectively. *Abbreviations: Cr—Creatinine; PCr—Phosphocreatinine; Glu—Glutamate; Gln—Glutamine; Ins—Myo-inositol; Tau—Taurine; Cho—Choline; PCh—Phosphocoline; GPC—Glycerophosphocholine; GSH—Glutathione; NAA—N-acetylaspartate; NAAG—N-acetylaspartylglutamate; GABA—Gamma-Amino Butyric Acid; Lac—lactate; MM—Macromolecules; Lip—Lipids. Image created by me or a co-author*

Interestingly, in MJD patients, GM cerebellar volume decreased significantly with increasing age of onset (*β* ± SE: − 0.54 ± 0.12, t = 4.43, p < 0.001) and SARA scores (*β* ± SE: − 0.50 ± 0.16, t = 3.24, p < 0.01), whereas in the WM cerebellar volume, no effect of these variables was observed (Additional file 5: Supp. Figures 5A and B). Pericerebellar CSF spaces (i.e., CSF cerebellar volume) increase significantly with SARA scores (*β* ± SE: 0.29 ± 0.10, t = 2.87, p < 0.05), but not with age of onset (*β* ± SE: 0.12 ± 0.08, t = 1.54, p = 0.15) (Additional file 5: Supp. Figure 5C). On the contrary, total cerebellar volume decreased significantly with increasing age of onset (*β* ± SE: − 0.61 ± 0.21, t = 2.93, p < 0.05), but not with SARA scores (*β* ± SE: − 0.17 ± 0.29, t = 0.65, p = 0.53) (Additional file 5: Supp. Figure 5D).

### II—Similarities in neurochemical alterations found in MJD transgenic mice (Tg-ATXN-3-69Q) and MJD patients

To further investigate neurometabolic changes in MJD cerebellum, ^1^H-MRS studies were performed in WT and MJD mice (28 mice/group, including mice of 2, 4, and 16 months old, Fig. 2A and B) and in two MJD patients (one pre-symptomatic and one symptomatic) and two gender age-matched controls (Fig. 2C and D) (see Additional file 11: Supp. Table 2). Average plots showing the concentration of metabolites in the cerebellum of MJD mice (Fig. 2B) and patients (Fig. 2D) were obtained, denoting differences in several neurometabolites.

**Fig. 3:**
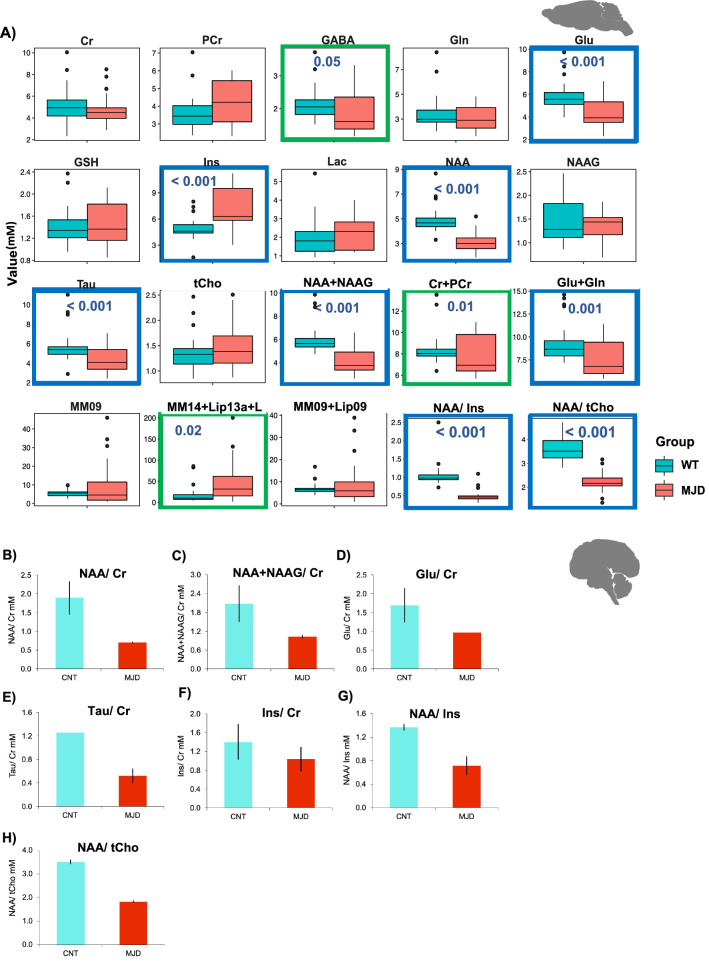
Neurochemical alterations in MJD mice and patients. **a** Boxplots (median, 25–75% inter-quartile range, non-outlier range, and outliers) illustrating concentration of neurometabolites in the cerebellum of WT and MJD mice (all ages included). **b–h** Graphic representation of the concentration (mM) of the principal cerebellar neurometabolies in MJD patients (n = 2) and gender and age-matched controls (CNT, n = 2): NAA (**b**), NAA + NAAG (**c**), Glu (**d**), Tau (**e**) and Ins (**f**), normalized with Cr; as well as the ratios NAA/Ins (**g**) and NAA/Total Cho (**h**). *In (a), Bonferroni corrected t-tests were used to test for differences among groups (WT, n* = *28; MJD, n* = 28). *p < 0.05, **p < 0.01, and ***p < *0.001. Abbreviations: Cr—Creatinine; PCr—Phosphocreatinine; Glu—Glutamate; Gln—Glutamine; Ins—Myo-inositol; Tau—Taurine; tCho—total Choline (Phosphocoline* + *Glycerophosphocholine); GSH—Glutathione; NAA—N-acetylaspartate; NAAG—N-acetylaspartylglutamate; GABA—Gamma-Amino Butyric Acid; Lac—lactate; MM—Macromolecules; Lip—Lipids. Image created by me or a co-author*

Principal Component Analysis (PCA) of neurochemicals in mice revealed a clear distinct subpopulation for WT (blue dots) and MJD mice (red dots) (Fig. 2E). Most relevant metabolites of PC1 and PC2 were then depicted from PCA analysis (Additional file 6: Supp. Figure 6), and their concentrations in the cerebellum of WT and MJD mice compared. The following four neurochemicals were significantly decreased (p < 0.001) in MJD cerebella regardless of age: N-acetylaspartate (NAA, 0.71 ± 0.22 *vs.* 1.02 ± 0.25, F_1,54_ = 13.54, p < 0.001), NAA + N-acetylaspartylglutamate (NAA_NAAG, 0.91 ± 0.27 *vs.* 1.24 ± 0.26, F_1,54_ = 16.04, p < 0.001), Glutamate (Glu, 4.29 ± 1.25 vs 5.80 ± 1.27, F_1,54_ = 12.24, p < 0.001) and Taurine (Tau, 4.35 ± 1.22 *vs.* 5.73 ± 1.66, F_1,54_ = 7.01, p < 0.001), whereas myo-Inositol (Ins, 6.97 ± 2.32 *vs.* 4.95 ± 1.25, F_1,54_ = 23.01, p < 0.001) was increased (Fig. 3A). Macromolecules (MM) and Lipids (Lip) were also increased in MJD mice (MM14_Lip13a_L, F_1,54_ = 6.09, P = 0.02). Moreover, neurochemical ratios, which are typically used in clinical assessments in SCAs, such as NAA/Ins [31] and NAA/tCho ratios [20], were decreased in this mouse model (NAA/Ins ratio: 0.48 ± 0.15 *vs.* 1.04 ± 031, F_1,54_ = 29.33, p < 0.001 and NAA/ tCh ratio: 2.22 ± 0.39 *vs.* 3.65 ± 0.50, F_1,54_ = 34.94, p < 0.001) (Fig. 3A). The same analysis was repeated by normalizing the concentration of each neurometabolite by creatine levels. No major differences were detected when using raw or normalized data (Additional file 7: Supp. Figures 7A–C).

A cross-sectional study to assess neurochemical cerebellar changes over time was also performed between WT and MJD mice. No major differences were found in this model, with exception for Glutamate and Ins (Additional file 8: Supp Figs. 8A to E). Glutamate was only decreased at later stages (Glu: 16 months-old: F_5,50_ = 13.48, p < 0.0001; WT: 5.01 ± 0.23, n = 7 vs MJD: 3.52 ± 0.25, n = 6, p = 0.05) (Additional file 8: Supplemental Fig. 8C), whereas Inositol only showed initial increase (Ins: 2 months old: F_5,49_ = 7.157, p < 0.0001; WT: 5.42 ± 0.31, n = 14 vs MJD: 38.08 ± 0.65, n = 14, p = 0.0008) (Additional file 8: Supp. Figures 8D), suggesting that Glutamate and Ins can be pointed as late and early markers of the disease, respectively, in this mouse model.

Importantly, in exploratory MRS acquisitions performed in MJD patients, the profiles of the key neurometabolites NAA, NAA + NAAG, Glu and Tau (Fig. 3B to H) were like those observed for the Tg-ATXN-3-69Q model (Fig. 3A) (Additional file 11: Supp. Table 2). Ins was the only candidate marker that did not vary in the same way as in the Tg-ATXN-3-69Q MJD mice (Fig. 3F). Finally, the NAA/Ins and NAA/tCho ratios presented clear distinct patterns between MJD and controls (Fig. 3G and H) in both pre and symptomatic stages (Additional file 11: Supp. Table 2), thus suggesting that these neurochemicals and their ratios can probably be used as disease biomarkers. In any case, the number of individuals analysed was reduced and needs to be validated with a larger number of MJD patients.

### III—Levels of key cerebellar neurochemicals are intrinsically associated to motor behaviour in transgenic MJD mice (Tg-ATXN-3-69Q model)

**Fig. 4:**
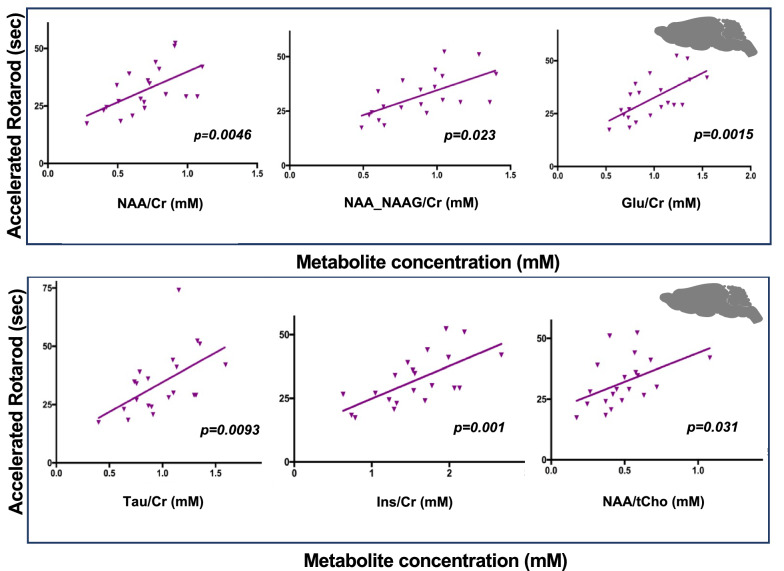
Relationship between the cerebellar concentration of key metabolites and accelerated rotarod performance on Tg-ATXN3-69Q (MJD) Mice. *Linear regression* between the cerebellar concentration (mM) of NAA, NAA + NAAG, Glu, Tau and Ins normalized with Cr, as well as NAA/tCho ratio, and the performance of MJD mice of 2 and 4 months of age (n = 22) in accelerated rotarod (sec). *p < 0.05 and ***p < *0.001. Abbreviations: NAA—N-acetylaspartate; NAAG—N-acetylaspartylglutamate; Cr—Creatinine; Glu—Glutamate; Tau—Taurine; Ins—Myo-inositol; tCho—Total Choline. Image created by me or a co-author*

Next, linear regressions between the rotarod and levels of five main altered cerebellar neurometabolites in MJD mice (NAA, NAA + NAAG, Glu, Tau, and Ins) were performed. The rotarod study was only carried out in MJD mice of 2 and 4 months of age, as older animals (16month-old) presented already some loss of strength in their limbs. Moreover, since no differences in accelerated and stationary rotarod were observed between MJD mice of 2 and 4 months-old (Additional file 9: Supp. Figure 9A and B), these two age groups were included in the same correlation analysis (Fig. 4).

There was a positive linear correlation between accelerated rotarod performance and levels of NAA/Cr (r^2^ = 0.3519, p = 0.0046), NAA + NAAG/Cr (r^2^ = 0.3950, p = 0.0023), Glutamate/Cr (r^2^ = 0.59, p = 0.0015), Tau/Cr (r^2^ = 0.4123, p = 0.0093), Ins/Cr (r^2^ = 0.4437, p = 0.0010) and the ratio between NAA and total Cho (NAA/total Cho) (r^2^ = 0.2219, p = 0.0311) (Fig. 4). Linear regressions for stationary rotarod were also performed and showed similar tendencies (Additional file 9: Supp. Figure 9C).

These results validate the use of ^1^H-MRS for the evaluation of the phenotypical status of this transgenic mouse model of MJD.

## Discussion

Structural MRI and ^1^H-MRS are powerful non-invasive techniques to determine brain alterations in SCAs [32], but studies in animals are scarce. Importantly, the equipment and methodology of MRI/MRS in mice and humans are highly similar providing an opportunity for direct translation of neuronal imaging biomarkers with accuracy and relevance. This study shows for the first time that structural and spectroscopic cerebellar modifications in a severe transgenic mouse model of MJD correlate with mice motor performance and with MJD patients’ data.

An essential step to identify translatable biomarkers is to first characterize mouse models that mimic human patients’ diseases [31]. For this purpose, we performed a thorough characterization of both anatomical and neurochemical cerebellar profiles in a severely impaired transgenic MJD mouse model, the Tg-69Q-ATXN3 mouse and further correlated with data collected from human patients. An extensive cerebellar volume loss was detected from early stages in Tg-69Q-ATXN3 mice and in MJD patients, accompanied by decrease of both cerebellar white and grey matter, and a clear increase of the fourth ventricle volume. These observations are in accordance with previous human MJD patients reports over atrophy of the cerebella [33–35], changes of white matter microstructure [36–38], as well as enlargement of the fourth ventricle documented from the initial disease stages [29, 30]

Machado-Joseph disease causes chronic axonal damage and extensive myelin degeneration in MJD [39]. Accordingly, we found pronounced loss of both white and grey matter in the cerebellum, irrespectively of the gender in human patients [37]. Though a decrease in grey matter was also found in normal controls, indicating that losses also occur with natural ageing, cerebellar atrophy was more profound in MJD patients, and inversely correlated with age of onset and disease severity, in accordance with previous reports [21, 30, 34, 40, 41]. However, these correlations were not observed in white matter volumetric assessments. White matter abnormalities in cerebellar peduncle develop early in disease progression and are even present in pre-symptomatic stages [37]. In fact, it has been proposed that myelin dysfunction/oligodendrocyte impairments occur previously to axonal degeneration and neuronal cell death in MJD [42]. Likewise, diffusion tensor imaging (DTI) abnormalities have been found in cerebellar white matter tracts of asymptomatic MJD gene carriers [39]. These studies highlight the importance of assessing white matter when performing neuronal imaging assessments, either in humans or in animal models. Here, Tg-69Q-ATXN3 mice showed, in accordance with human MJD data, cerebellar white matter loss. However, whether white matter starts to degenerate early, mimicking the human disease pathophysiology, remains to be elucidated since we only computed white matter at advanced ages. In future studies, we will assess white matter volume and DTI measurements at earlier timepoints to address this question. The fact that cerebellar white matter loss does not correlate with SARA scores and age of onset is intriguing and requires further evaluation. Indeed, abnormal DTI values correlated with SARA and ICARS scores in other studies [21], indicating that damage of the WM may be closely related with disease severity.

Regarding the fourth ventricle, we noted that CSF volume was incremented in male MJD patients, but this was not statistically significant in females. Consequently, the total cerebellar volume also changed differently according to the gender, being it lower in females than in males. Pre-existing studies observed enlargement of the fourth ventricle in MJD patients, but they do not report gender separation or included a small number of patients [29, 30]. Based on this, it seems important to validate this in a larger cohort. Similarly, gender influence on CSF volume should be assessed in future studies.

Importantly, ventricle enlargement was positively correlated with SARA score, highlighting its relevance in disease severity. Therefore, if females are proven to show CSF volume increments with disease progression in larger cohorts, this may be a promising disease marker.

Another set of biomarkers that have been used to characterize SCAs are neurochemicals quantified through ^1^H-MRS. Having in consideration that alterations in the levels of five key neurometabolites (NAA, NAA + NAAG, Glutamate, Taurine and Ins) in the cerebellum of the studied MJD mouse model were detected from the age of two months, we can conclude that in this model, neurometabolic alterations are established very early and are extremely severe, denoting extensive neuronal loss mirrored by low levels of total NAA and neurotransmitters Glutamate and Taurine, in accordance with the neurochemical profile as previously observed for YAC MJD mice [23, 24].

On the other hand, the increased levels of Ins observed MJD mice, similarly to humans, is suggestive of an initial activation of microglia, which may be neuroprotective, in this way compensating the lack of functional neurons [19] and contributing to preservation of motor performance. Nonetheless, Ins concentration may vary between different models since in the YAC model, there was a reduction in Ins cerebellar levels [24]. It is though hard to establish a direct comparison between different models with disparate levels of severity and pathological characteristics, that even with identical ages might correspond to different disease grades. This variation may be also present in human patients according to the stage of the disease at the time of MRS studies and must thus be assessed carefully [43]. Accordingly, in our exploratory ^1^H-MRS study, the two MJD patients assessed here presented either a reduction (pre-symptomatic stage), or maintenance of the levels of Ins (symptomatic stage), unlike previous reports in humans [19]. This of course requires further assessments to come to assertive conclusions. Nonetheless, our mouse model results are supported by observations in MJD patients in previous studies, namely the lower NAA associated with concomitant higher concentrations of Ins in the cerebellar vermis, cerebellar white matter and in the pons [19, 43, 44].

Unfortunately, there are still no defined markers (or a set of markers) specific to MJD. Despite the efforts of using spatiotemporal signatures for different SCAs [44, 45], all these markers here referred or pointed out in other studies are common to many degenerative disorders, since they reflect glial disturbances, axonal loss and degeneration of neurons, neurotransmission impairments and neuroinflammation. However, MJD is a genetic known disorder that does not require the use of a neurometabolic profile for its diagnosis, but otherwise a set of markers that allow for the clinical evaluation of disease stage. In that sense, the neurochemical ratio NAA/total Cho, which is indicative of brain metabolism and was previously correlated with clinical status in SCA patients [21, 46, 47] was also reproduced both in Tg-ATX3-69Q mice and MJD patients, suggesting that this animal model has clinical relevance and is suitable for evaluating neurochemical alterations driven by putative therapies. Since it is a severe model, it probably mimics a late clinical stage. Even though the levels of Cho and Ins alone show some inter-studies variability in human patients, future assessments of both neurochemicals at different stages of the disease may add interesting information regarding this aspect, and could indicate Cho and Ins as good indicators of disease severity [43].

Despite the fact this model did not show longitudinal changes given the acute severity from early time points on, we found the levels of Glutamate to worsen in later stages of the disease (16 months of age). In fact, this marker was found to be decreased in the vermis and pons of MJD patients, even though to a lesser extent than NAA, indicating that Glutamate could also probably be a good candidate to be used as disease progression marker [19]. Supporting the theory that Glutamate can also serve as disease marker, we have recently shown that Glutamax (Glu + Gln) could be used to detect disease reversion in this model upon a treatment with mesenchymal stromal cells [11].

Importantly, in the present study the levels of neurochemicals NAA, NAA + NAAG, Glutamate, Taurine and Ins were significantly correlated with motor performance of Tg-Q69-ATXN3 mice, as assessed by rotarod. Similarly, previous studies in SCA1 models have reported changes in NAA, Ins and Glutamate, which also correlated with disease stage evaluated by molecular layer thickness measurements and overall severity score [48]. In addition, these neurospectroscopic markers have also been correlated with patients’ severity according to ataxia scores [49]. More recently, the ratio NAA/Ins was suggested to be indicative of disease severity in (conditional) SCA1 mice [31]. In our study, this ratio was severely diminished in Tg-69Q-ATXN3 mice at all ages, besides the clear tendency to also be decreased in pre- or post-symptomatic patients.

Therefore, the neurochemicals that correlate with clinical status in MJD patients also showed to be important biomarkers in the Tg-69Q-ATXN3 mouse model, and this characterization may thus serve in future assessments of therapies’ efficiency in the same MJD mouse model.

## Limitations of this study

As an obvious limitation of this study, the number of MJD patients included for ^1^H-MRS analysis is too low, since due to the COVID-19 pandemic we could not continue recruiting more volunteers, and some had to be cancelled. This study also lacks gender match in 4 and 16 months-old animal groups. Though it would be important to have age-match groups at all ages, we have analyzed the rotarod performance of male and female mice separately at 2 months, and no sex differences were found (data not shown), which possibly minimizes the concerns of lacking this balance. In future studies it would be interesting to perform neuropathological assessments in mice and post-mortem tissue to correlate with reported neurochemical alterations. Despite these limitations, this study points for a set of valid biomarkers that can be valuable in the clinics.

## Conclusion

MRI/MRS techniques are highly relevant to track disease progression and application of such practices to animal models is necessary to get maximum profit from disease models [50, 51]. We found that MRI/MRS-based non-invasive biomarkers of a severely impaired MJD transgenic mouse model are similar to those found in patients and correlate with motor performance in mice. Thus MRI/MRS biomarkers can be used as a readout in preclinical trials of promising therapeutic agents in MJD mice and subsequently translated to human clinical trials contributing to the identification of successful therapies to MJD. In the future, it would be important to perform longitudinal studies, i.e., repeated measurements in the same animal at different age (perhaps in another model with later disease onset), as well as in patients, in order to establish true evolutive curves of these principal neurochemicals.

## Supplementary Information

**Additional file 1**. Segmentation of cerebellar WM/GM. Column A: automatic WM/GM edge detection (green lines) based on Laplacian of Gaussian method; Column B: automatic ROIs identification; Column C: WM identification (green regions) after manual ROIs selection. Image created by me or a co-author. **Additional file 2**. Correlations between independent variables. When two variables were highly correlated (r > 0.7), only the variable with the lowest Akaike Information Criteria (AIC) and p value in a univariate model was selected, to avoid collinearity issues. Image created by me or a co-author. **Additional file 3**. Timeline of the experimental procedure in animals. (A) In the present study, 28 wild type (WT) and 29 Tg-ATXN3-69Q (MJD) mice were studied. At 2 months of age, 14 WT animals and 14 MJD mice performed behavioural studies (rotarod), their cerebella were analysed by in vivo Magnetic Resonance Imaging/Proton-Magnetic Resonance Spectroscopy (MRI/1H-MRS) and were sacrificed. At 4 months of age, 7 WT and 9 MJD mice were subjected to the same procedures and then sacrificed. Finally, at 16 months of age, 7 WT and 6 MJD mice were analysed by MRI/ 1H-MRS and sacrificed. Image created by me or a co-author. **Additional file 4**. Comparison of cerebellar volume at three different ages (2, 4 and 16 months) in WT and MJD mice. Cerebellar volume (mm^3^) of WT and MJD mice obtained through the analysis of cerebellar images pre-processed and segmented in Matlab R2012b at 2 (n=14 WT vs n=14 MJD), 4 (n=7 WT vs n=9 MJD) and 16 months of age (n=7 WT vs n=6 MJD). One-way ANOVA, followed by Tukey’s multiple comparisons test to test for differences among groups, ***p < 0.001. Image created by me or a co-author. **Additional file 5**. Multiple regression tests of covariates on MJD patients (n=16). Multiple regressions were used to test the effects of age of onset and SARA scores on GM, WM, CSF, and total cerebellar volumes. Disease duration and number of CAG repeats in disease allele were removed from the analysis because of high collinearity (r > 0.7). Image created by me or a co-author. **Additional file 6**. Correlation of metabolites with the two first axis of a Principal Component Analysis 1 (PC1) and 2 (PC2) of the cerebellar neurometabolites analysed by ^1^H-MRS in WT and MJD mice (raw data). Metabolites with higher correlation with PC1 and PC2 highlighted by the blue box in WT (n=28) and Tg-ATXN3-69Q (MJD) mice (n=28), when considering raw data. Abbreviations: Cr – creatinine; PCr – phosphocreatinine; Glu – glutamate; Gln – glutamine; Ins– myo-inositol; Tau – taurine; Cho – choline; PCh – phosphocoline; GPC - glycerophosphocholine; GSH – glutathione; NAA – N-acetylasparate; NAAG – N-acetylaspartylglutamate; GABA - Gamma-Amino Butyric Acid; Lac – lactate; MM - macromolecules; Lip - lipids. Image created by me or a co-author. **Additional file 7**. Normalized data of cerebellar Proton Magnetic Resonance Spectroscopy assessed in WT and MJD mice. (A) Principal Component Analysis (PCA) of the neurometabolites analysed by 1H-MRS and normalized with total Creatine in WT and Tg-ATXN3-69Q (MJD) mice, branched in PC1 and PC2 (corresponding to 49.3% and 16.2% explaining variables, respectively). (B) Metabolites with higher correlation with PC1 and PC2 highlighted by the blue box in WT and MJD mice, when considering values normalized with total Creatine. (C) Boxplots (median, 25–75% inter-quartile range, non-outlier range, and outliers) illustrating concentration of neurometabolites in the cerebellum of WT and MJD mice, when considering values normalized with total Creatine (all ages included). A blue and green box is surrounding the metabolites with significant different concentrations between groups in a PERMANOVA analysis (p<0.001 and p≤0.05, respectively). Bonferroni corrected t-tests were used to test for differences among groups (WT, n=28; MJD, n=28). ***p <0.001. Abbreviations: Cr – Creatinine; PCr – Phosphocreatinine; Glu – Glutamate; Gln – Glutamine; Ins – Myo-inositol; Tau – Taurine; tCho – total Choline (Phosphocoline+Glycerophosphocholine); GSH – Glutathione; NAA – N-acetylasparate; NAAG – N-acetylaspartylglutamate; GABA - Gamma-Amino Butyric Acid; Lac – Lactate; MM - Macromolecules; Lip - Lipids. Image created by me or a co-author. **Additional file 8**. Comparison of cerebellar levels of key neurometabolites at three different ages (2, 4 and 16 months) in WT and MJD mice. (A-E) Concentration (mM) of principal cerebellar neurometabolites, previously identified through PCA analysis of WT and MJD mice (NAA, NAAG, Glutamate, Ins and Taurine) at 2, 4 and 16 months of age. One-way ANOVA, followed by Tukey’s multiple comparisons test to test for differences among groups (WT, n=28; MJD, n=28). p < 0.05, **p < 0.01. Abbreviations: Cr – Creatinine; Glu – Glutamate; Ins – Myo-inositol; tCho – Total choline; NAA – N-acetylasparate; NAAG – N-acetylaspartylglutamate. Image created by me or a co-author. **Additional file 9**. Relationship between the cerebellar concentration of key metabolites and rotarod performance on Tg-ATXN3-69Q (MJD) Mice. (A-B) Coordination and balance assessed through accelerated (A) and stationary rotarod (B) of MJD mice at 2 and 4 months of age. Unpaired t test with Welch’s correction was performed (MJD 2M, n=14; MJD 4M, n=8). (C) Linear regression between the concentration (mM) of NAA, NAA+NAAG, Glu, Tau and Ins normalized with Cr, as well as NAA/tCho ratio, and the stationary rotarod performance of MJD mice (n= 22), for significance, *p < 0.05. Abbreviations: NAA – N-acetylasparate; NAAG – N-acetylaspartylglutamate; Glu – Glutamate; Tau – Taurine; Ins– Myo-inositol; tCho – Total choline; Cr – Creatinine. Image created by me or a co-author. **Additional file 10**. Patients cohort for MRI volumetric assessments. Abbreviations: MJD – Machado-Joseph disease or Spinocerebellar ataxia type 3; CNT- control; SARA - Scale for the assessment and rating of ataxia; CAGexp- expanded allele; CAGnorm – normal allele; 0=missing information. Image created by me or a co-author. **Additional file 11**. 1H-MRS values (mM), normalized with creatine for the key neurochemicals or neurochemical ratios. Abbreviations: MJD – Machado-Joseph disease/spinocerebellar ataxia type 3; CNT – controls; SARA - Scale for the assessment and rating of ataxia; CAGexp- expanded allele; NAA – N-acetylasparate; Cr – creatinine; NAAG – N-acetylaspartylglutamate; Glu – Glutamate; Tau – Taurine; Ins – Myo-inositol; Cho – Choline; tCho – Total choline; ND – not determined. Image created by me or a co-author.

## Data Availability

The authors confirm that the data supporting the findings of this study are available within the article and its supplementary materials. Other data that support the findings of this study are available from the corresponding authors, [COM, RJN or LPA], upon reasonable request.
