# Supplementary material for: Cerebellar morphometric and spectroscopic biomarkers for Machado-Joseph Disease
Source: Acta Neuropathol Commun. 2022 Mar 19;10:37. doi: 10.1186/s40478-022-01329-4 (PMC8933766; doi:10.1186/s40478-022-01329-4)
Supplement: Supplementary file 1 — Additional file 1. Segmentation of cerebellar WM/GM. Column A: automatic WM/GM edge detection (green lines) based on Laplacian of Gaussian method; Column B: automatic ROIs identification; Column C: WM identification (green regions) after manual ROIs selection. Image created by me or a co-author. Additional file 2. Correlations between independent variables. When two variables were highly correlated (r > 0.7), only the variable with the lowest Akaike Information Criteria (AIC) and p value in a univariate model was selected, to avoid collinearity issues. Image created by me or a co-author. Additional file 3. Timeline of the experimental procedure in animals. (A) In the present study, 28 wild type (WT) and 29 Tg-ATXN3-69Q (MJD) mice were studied. At 2 months of age, 14 WT animals and 14 MJD mice performed behavioural studies (rotarod), their cerebella were analysed by in vivo Magnetic Resonance Imaging/Proton-Magnetic Resonance Spectroscopy (MRI/1H-MRS) and were sacrificed. At 4 months of age, 7 WT and 9 MJD mice were subjected to the same procedures and then sacrificed. Finally, at 16 months of age, 7 WT and 6 MJD mice were analysed by MRI/ 1H-MRS and sacrificed. Image created by me or a co-author. Additional file 4. Comparison of cerebellar volume at three different ages (2, 4 and 16 months) in WT and MJD mice. Cerebellar volume (mm3) of WT and MJD mice obtained through the analysis of cerebellar images pre-processed and segmented in Matlab R2012b at 2 (n=14 WT vs n=14 MJD), 4 (n=7 WT vs n=9 MJD) and 16 months of age (n=7 WT vs n=6 MJD). One-way ANOVA, followed by Tukey’s multiple comparisons test to test for differences among groups, ***p < 0.001. Image created by me or a co-author. Additional file 5. Multiple regression tests of covariates on MJD patients (n=16). Multiple regressions were used to test the effects of age of onset and SARA scores on GM, WM, CSF, and total cerebellar volumes. Disease duration and number of CAG repeats in disease allele were removed fr [file 40478_2022_1329_MOESM1_ESM.pdf]

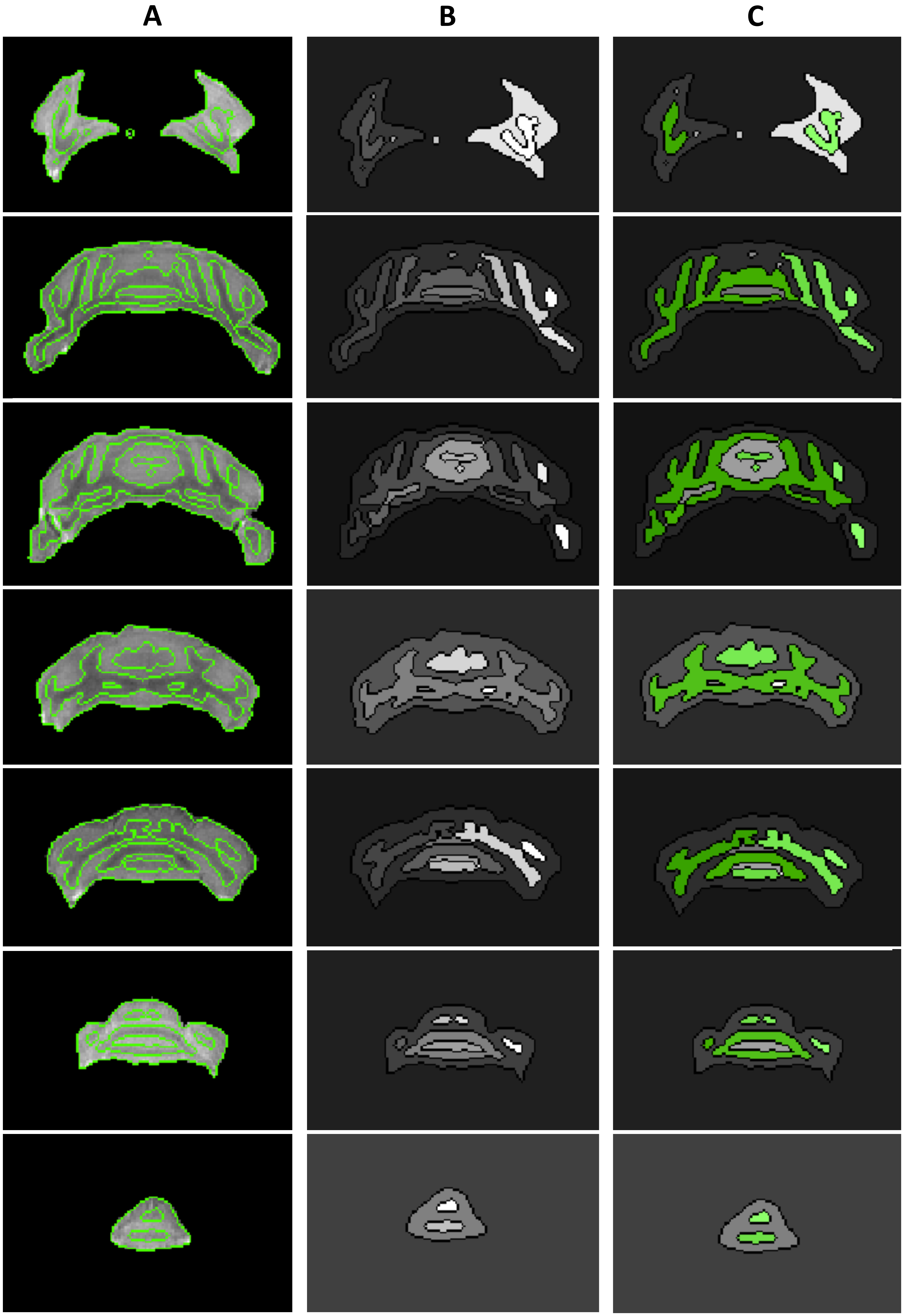

Additional file 1

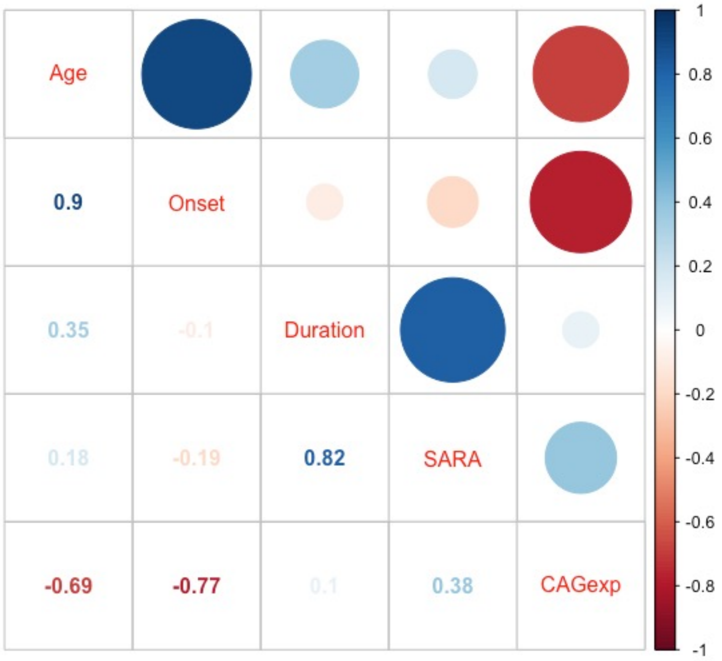

Additional file 2

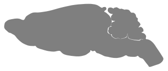

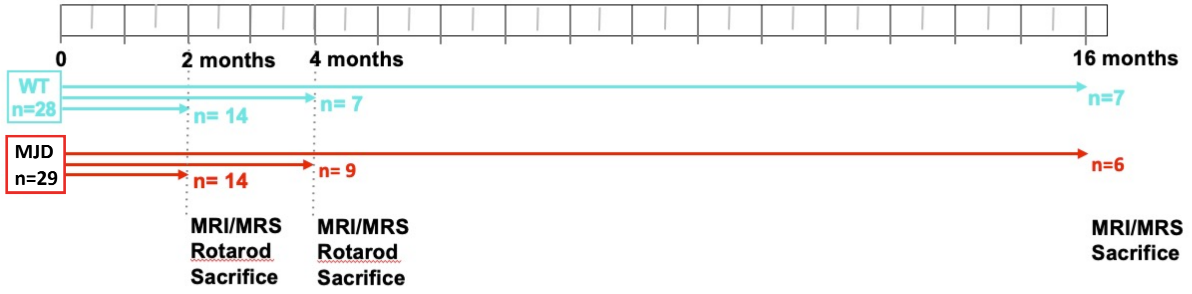
0
2 months
4 months
16 months
WT
n=28
n= 14
n= 7
n=7
MJD
n=29
n= 14
n= 9
n=6
MRI/MRS
Rotarod
Sacrifice
MRI/MRS
Rotarod
Sacrifice
MRI/MRS
Sacrifice

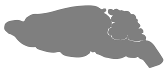

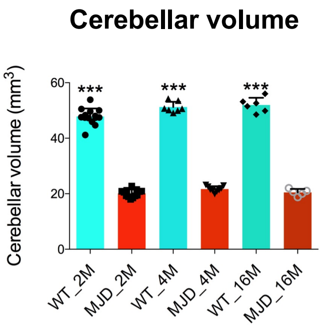

Additional file 4

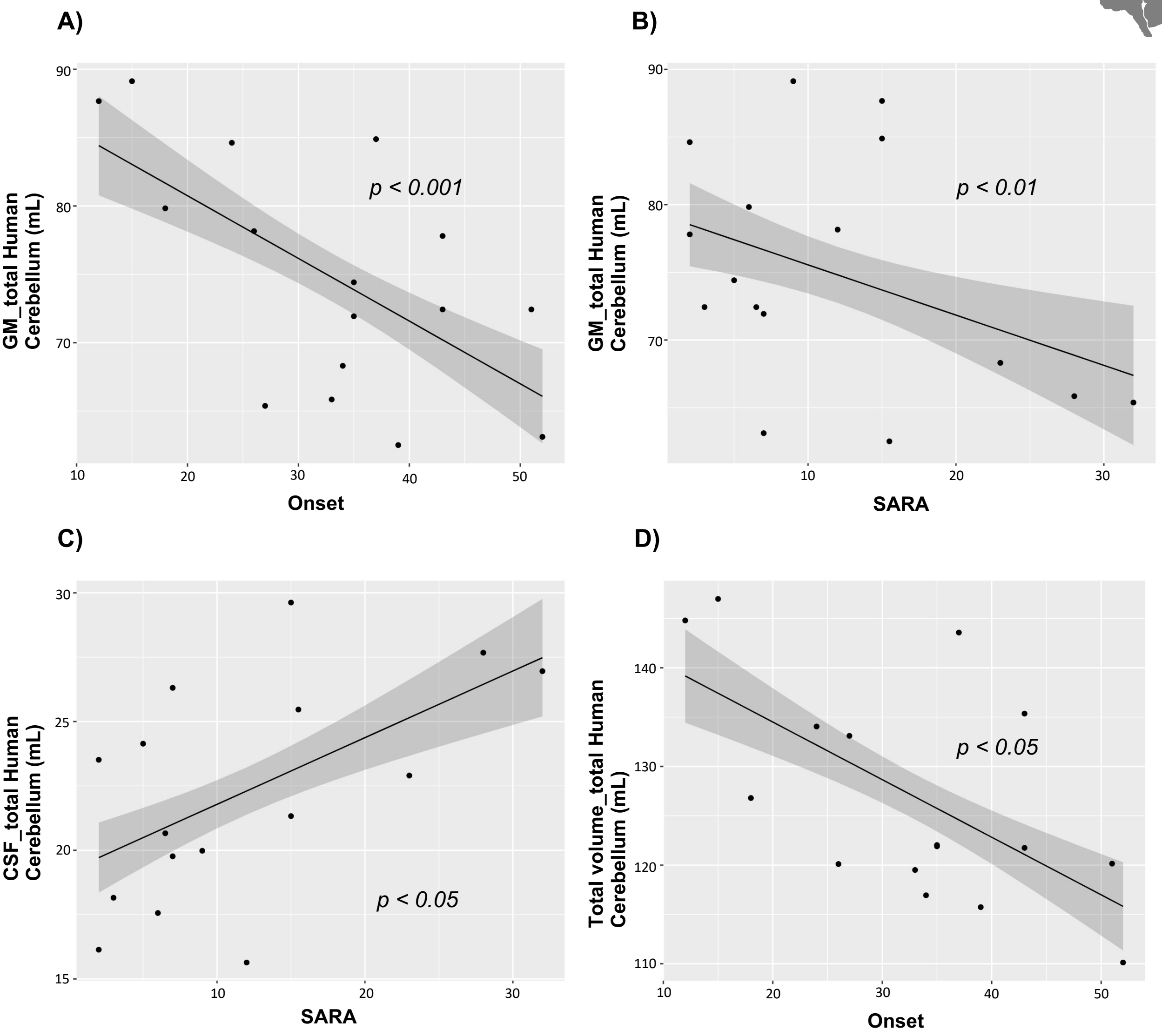

Additional file 5

A)

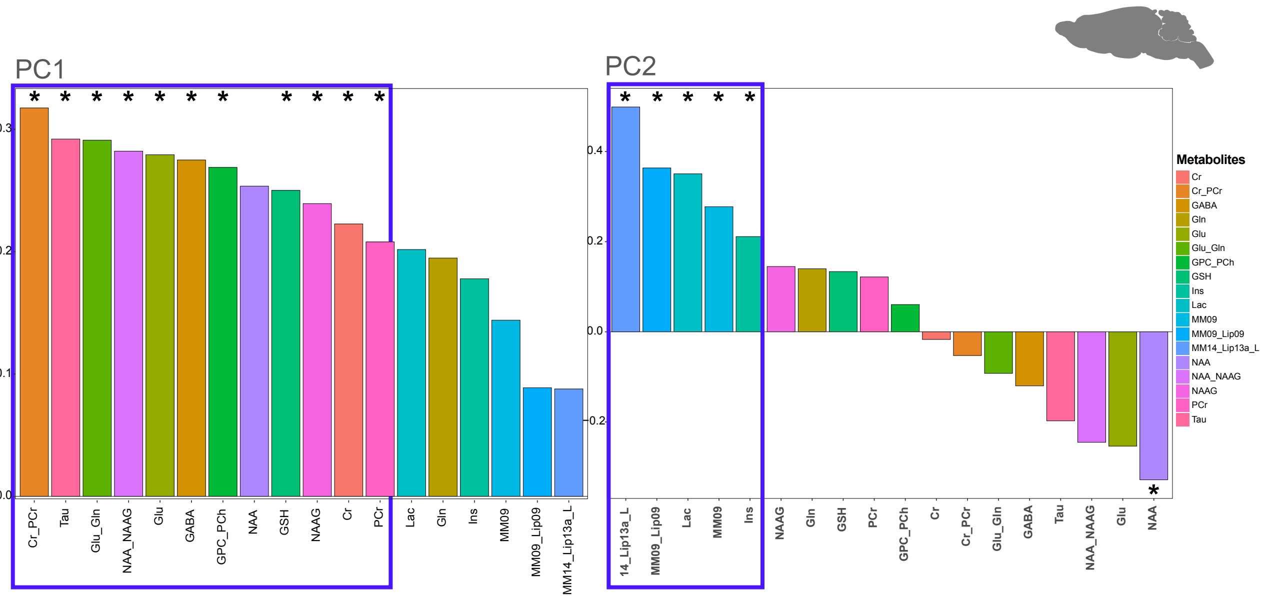

Additional file 6

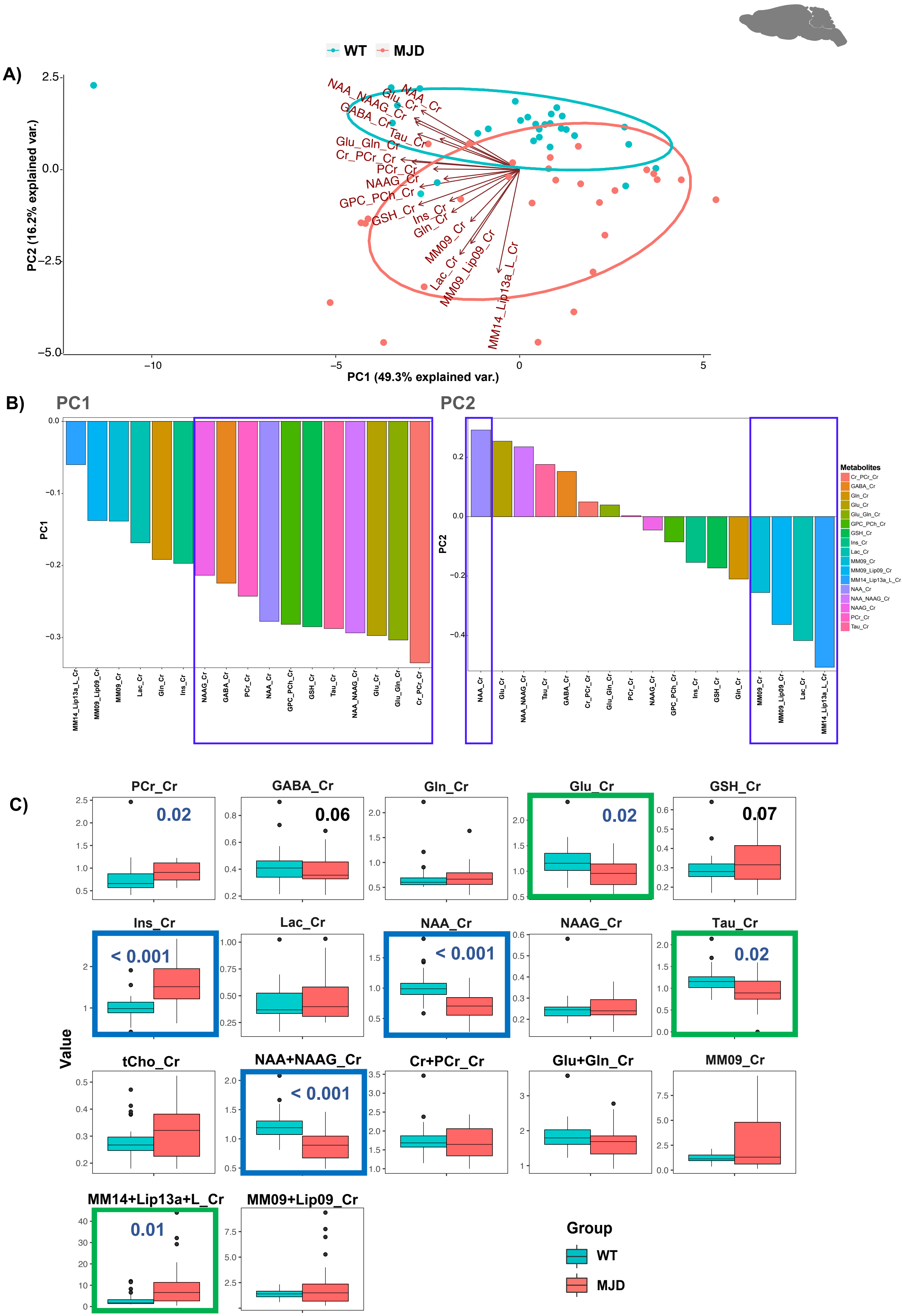

Additional file 7

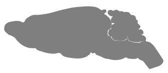

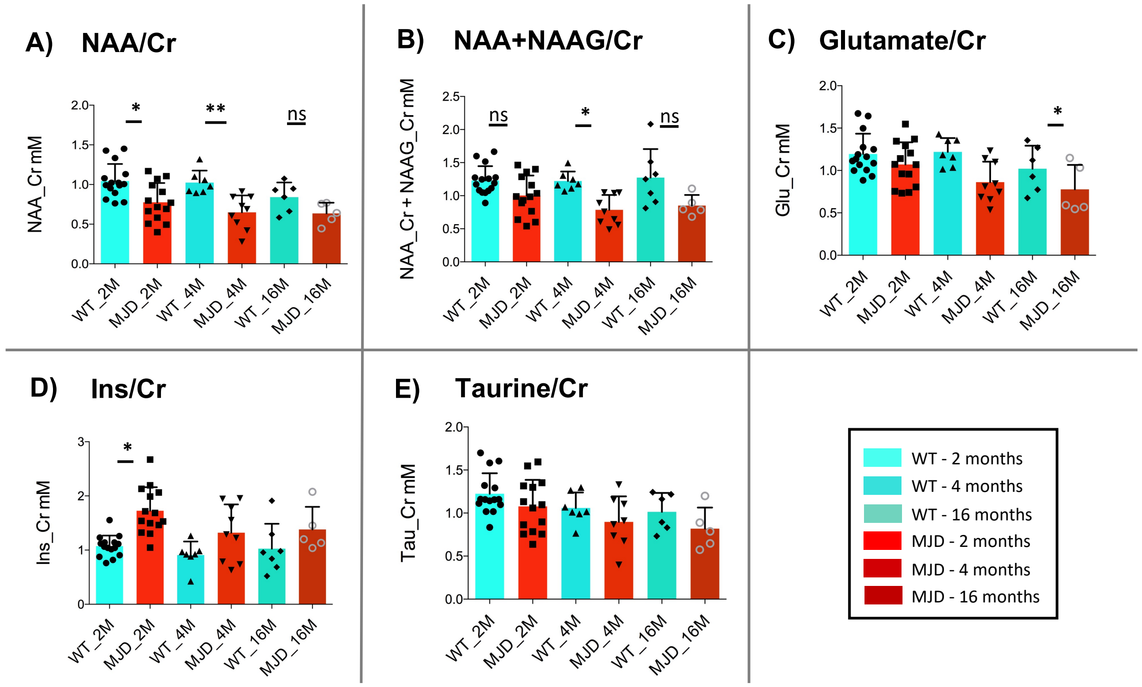

Additional file 8

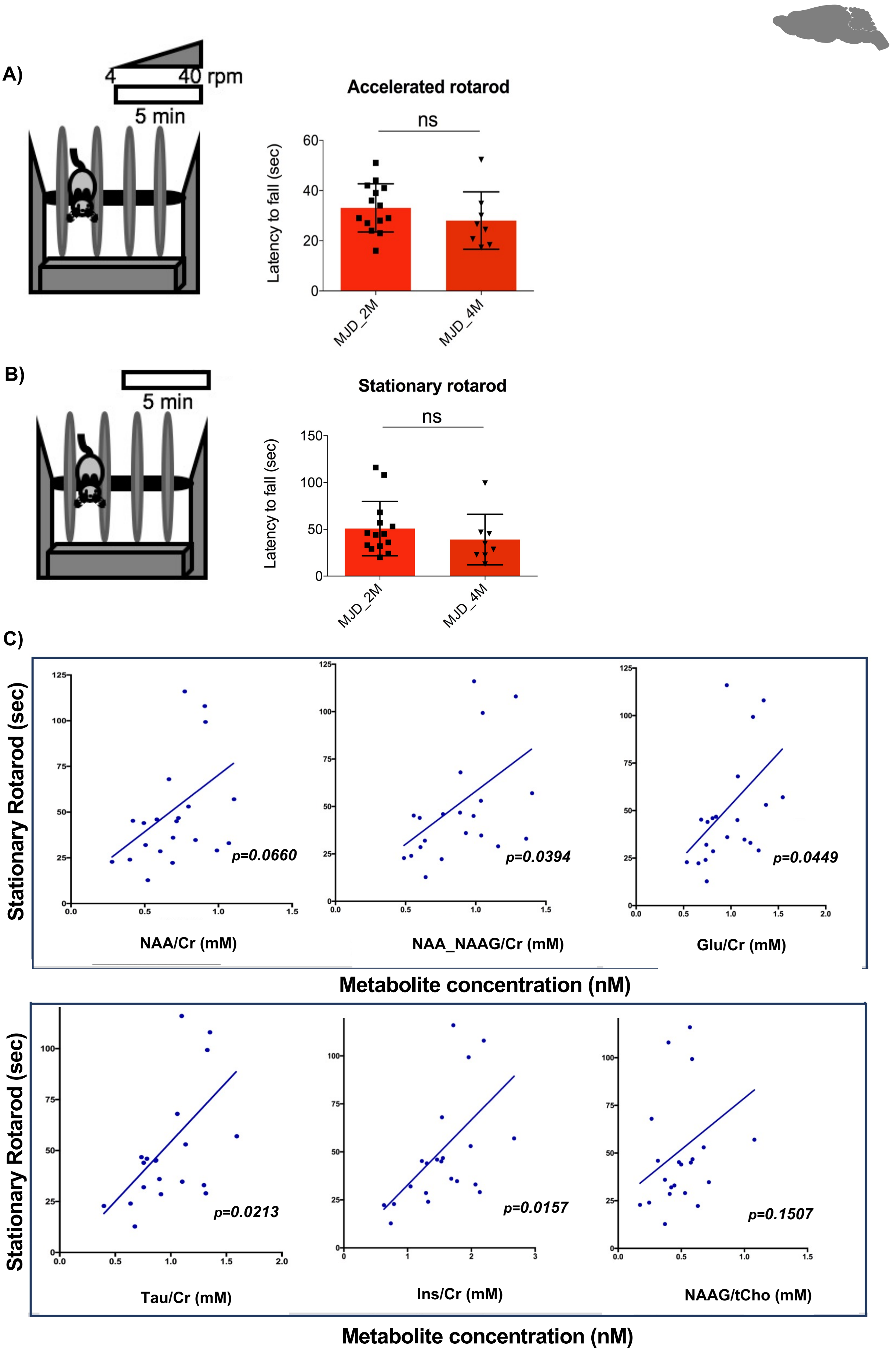

Additional file 9

Subject ID	Group	Gender	Age	Age at onset	Disease duration	SARA	CAGexp	CAGnorm
Subject 1	MJD/SCA3	Male	52.91	37	15.91	15	71	21
Subject 2	MJD/SCA3	Female	46.86	43	3.86	3	66	15
Subject 3	MJD/SCA3	Female	20.88	18	2.88	6	77	23
Subject 4	MJD/SCA3	Female	48.62	34	14.62	23	75	28
Subject 5	MJD/SCA3	Male	46.94	33	13.94	28	75	20
Subject 6	MJD/SCA3	Male	19.89	12	7.89	15	81	23
Subject 7	MJD/SCA3	Male	40.03	35	5.03	5	74	20
Subject 8	MJD/SCA3	Male	60.23	52	8.23	7	63	14
Subject 9	MJD/SCA3	Male	29.43	15	14.43	9	73	24
Subject 10	MJD/SCA3	Female	56.42	51	5.42	6.5	70	18
Subject 11	MJD/SCA3	Female	30.00	26	4	12	75	14
Subject 12	MJD/SCA3	Female	45.16	43	2.16	2	73	0
Subject 13	MJD/SCA3	Female	42.63	35	7.63	7	75	0
Subject 14	MJD/SCA3	Male	49.18	39	10.18	15.5	74	0
Subject 15	MJD/SCA3	Male	44.96	27	17.96	32	77	24
Subject 16	MJD/SCA3	Male	24.30	24	0.30	2	75	24
Subject 17	CNT	Male	24.51	-	-	-	-	-
Subject 18	CNT	Female	20.13	-	-	-	-	-
Subject 19	CNT	Female	27.69	-	-	-	-	-
Subject 20	CNT	Male	32.78	-	-	-	-	-
Subject 21	CNT	Male	22.92	-	-	-	-	-
Subject 22	CNT	Male	33.72	-	-	-	-	-
Subject 23	CNT	Male	30.85	-	-	-	-	-
Subject 24	CNT	Male	33.89	-	-	-	-	-
Subject 25	CNT	Female	47.72	-	-	-	-	-
Subject 26	CNT	Female	33.48	-	-	-	-	-
Subject 27	CNT	Male	24.90	-	-	-	-	-

Subject 28	CNT	Male	25.05	-	-	-	-	-
Subject 29	CNT	Female	57.62	-	-	-	-	-
Subject 30	CNT	Female	48.04	-	-	-	-	-
Subject 31	CNT	Female	27.75	-	-	-	-	-
Subject 32	CNT	Female	27.09	-	-	-	-	-
Subject 33	CNT	Male	33.20	-	-	-	-	-
Subject 34	CNT	Male	46.14	-	-	-	-	-

Subject ID	Gender	Age	Age at onset	Disease duration (years)	SARA score	CAG exp	NAA/ Cr	NAA+NAAG/ Cr	Glu/ Cr	Tau/ Cr	Ins/ Cr	tCho	NAA/ Ins	NAA/ tChol
MJD_Subject I	Female	32	-	0	3	65	0.682236	1.086564	ND	0.398196	0.779801	0.364112	0.874884	1.873699
MJD_Subject II	Female	56	42	16	6.5	66	0.725294	0.976176	0.971176	0.649411	1.295882	0.410000	0.559691	1.769010
CNT_Subject I	Female	57	-	-	-	-	1.457368	1.493224	1.248164	ND	1.021456	0.425748	1.426755	3.423076
CNT_Subject II	Female	33	-	-	-	-	2.339733	2.666927	2.157131	1.258620	1.782131	0.648119	1.312884	3.610036

Supplementary Information

Additional file 1. Segmentation of cerebellar WM/GM. Column A: automatic WM/GM edge detection (green lines) based on Laplacian of Gaussian method; Column B: automatic ROIs identification; Column C: WM identification (green regions) after manual ROIs selection. Image created by me or a co-author.

Additional file 2. Correlations between independent variables. When two variables were highly correlated ($r > 0.7$), only the variable with the lowest Akaike Information Criteria (AIC) and p value in a univariate model was selected, to avoid collinearity issues. Image created by me or a co-author.

Additional file 3. Timeline of the experimental procedure in animals. (A) In the present study, 28 wild type (WT) and 29 Tg-ATXN3-69Q (MJD) mice were studied. At 2 months of age, 14 WT animals and 14 MJD mice performed behavioural studies (rotarod), their cerebella were analysed by in vivo Magnetic Resonance Imaging/Proton-Magnetic Resonance Spectroscopy (MRI/^{1}H-MRS) and were sacrificed. At 4 months of age, 7 WT and 9 MJD mice were subjected to the same procedures and then sacrificed. Finally, at 16 months of age, 7 WT and 6 MJD mice were analysed by MRI/ ^{1}H-MRS and sacrificed. Image created by me or a co-author.

Additional file 4. Comparison of cerebellar volume at three different ages (2, 4 and 16 months) in WT and MJD mice. Cerebellar volume (mm^3) of WT and MJD mice obtained through the analysis of cerebellar images pre-processed and segmented in Matlab R2012b at 2 (n=14 WT vs n=14 MJD), 4 (n=7 WT vs n=9 MJD) and 16 months of age (n=7 WT vs n=6 MJD). One-way ANOVA, followed by Tukey's multiple comparisons test to test for differences among groups, ***$p < 0.001$. Image created by me or a co-author.

Additional file 5. Multiple regression tests of covariates on MJD patients (n=16). Multiple regressions were used to test the effects of age of onset and SARA scores on GM, WM, CSF, and total cerebellar volumes. Disease duration and number of CAG repeats in disease allele were removed from the analysis because of high collinearity ($r > 0.7$). Image created by me or a co-author.

Additional file 6. Correlation of metabolites with the two first axis of a Principal Component Analysis 1 (PC1) and 2 (PC2) of the cerebellar neurometabolites analysed by ^{1}H-MRS in WT and MJD mice (raw data). Metabolites with higher correlation with PC1 and PC2 highlighted by the blue box in WT (n=28) and Tg-ATXN3-69Q (MJD) mice (n=28), when considering raw data. Abbreviations: Cr – creatinine; PCr – phosphocreatinine; Glu – glutamate; Gln – glutamine; Ins– myo-inositol; Tau – taurine; Cho – choline; PCh – phosphocoline; GPC - glycerophosphocholine; GSH – glutathione; NAA – N-acetylasparate; NAAG – N-acetylaspartylglutamate; GABA - Gamma-Amino Butyric Acid; Lac – lactate; MM - macromolecules; Lip - lipids. Image created by me or a co-author.

Additional file 7. Normalized data of cerebellar Proton Magnetic Resonance Spectroscopy assessed in WT and MJD mice. (A) Principal Component Analysis (PCA) of the neurometabolites analysed by ^{1}H-MRS and normalized with total Creatine in WT and Tg-ATXN3-69Q (MJD) mice, branched in PC1 and PC2 (corresponding to 49.3% and 16.2% explaining variables, respectively). (B) Metabolites with higher correlation with PC1 and PC2 highlighted by the blue box in WT and MJD mice, when considering values normalized with total Creatine. (C) Boxplots (median, 25–75% inter-quartile range, non-outlier range, and

outliers) illustrating concentration of neurometabolites in the cerebellum of WT and MJD mice, when considering values normalized with total Creatine (all ages included). A blue and green box is surrounding the metabolites with significant different concentrations between groups in a PERMANOVA analysis ($p<0.001$ and $p\leq0.05$, respectively). Bonferroni corrected t-tests were used to test for differences among groups (WT, n=28; MJD, n=28). ***p <0.001. Abbreviations: Cr – Creatinine; PCr – Phosphocreatinine; Glu – Glutamate; Gln – Glutamine; Ins – Myo-inositol; Tau – Taurine; tCho – total Choline (Phosphocoline+Glycerophosphocholine); GSH – Glutathione; NAA – N-acetylasparate; NAAG – N-acetylaspartylglutamate; GABA - Gamma-Amino Butyric Acid; Lac – Lactate; MM - Macromolecules; Lip - Lipids. Image created by me or a co-author.

Additional file 8. Comparison of cerebellar levels of key neurometabolites at three different ages (2, 4 and 16 months) in WT and MJD mice. (A-E) Concentration (mM) of principal cerebellar neurometabolites, previously identified through PCA analysis of WT and MJD mice (NAA, NAAG, Glutamate, Ins and Taurine) at 2, 4 and 16 months of age. One-way ANOVA, followed by Tukey's multiple comparisons test to test for differences among groups (WT, n=28; MJD, n=28). $p < 0.05$, **$p < 0.01$. Abbreviations: Cr – Creatinine; Glu – Glutamate; Ins – Myo-inositol; tCho – Total choline; NAA – N-acetylasparate; NAAG – N-acetylaspartylglutamate. Image created by me or a co-author.

Additional file 9. Relationship between the cerebellar concentration of key metabolites and rotarod performance on Tg-ATXN3-69Q (MJD) Mice. (A-B) Coordination and balance assessed through accelerated (A) and stationary rotarod (B) of MJD mice at 2 and 4 months of age. Unpaired t test with Welch's correction was performed (MJD 2M, n=14; MJD 4M, n=8). (C) Linear regression between the concentration (mM) of NAA, NAA+NAAG, Glu, Tau and Ins normalized with Cr, as well as NAA/tCho ratio, and the stationary rotarod performance of MJD mice (n= 22), for significance, *$p < 0.05$. Abbreviations: NAA – N-acetylasparate; NAAG – N-acetylaspartylglutamate; Glu – Glutamate; Tau – Taurine; Ins– Myo-inositol; tCho – Total choline; Cr – Creatinine. Image created by me or a co-author.

Additional file 10. Patients cohort for MRI volumetric assessments. Abbreviations: MJD – Machado-Joseph disease or Spinocerebellar ataxia type 3; CNT- control; SARA - Scale for the assessment and rating of ataxia; CAGexp- expanded allele; CAGnorm – normal allele; 0=missing information. Image created by me or a co-author.

Additional file 11. ^{1}H-MRS values (mM), normalized with creatine for the key neurochemicals or neurochemical ratios. Abbreviations: MJD – Machado-Joseph disease/spinocerebellar ataxia type 3; CNT – controls; SARA - Scale for the assessment and rating of ataxia; CAGexp- expanded allele; NAA – N-acetylasparate; Cr – creatinine; NAAG – N-acetylaspartylglutamate; Glu – Glutamate; Tau – Taurine; Ins – Myo-inositol; Cho – Choline; tCho – Total choline; ND – not determined. Image created by me or a co-author.
